# Successful rescue of a high-risk pulmonary embolism using thrombolysis, extracorporeal membrane oxygenation, and percutaneous pulmonary thrombectomy: a case report

**DOI:** 10.3389/fcvm.2026.1800854

**Published:** 2026-03-25

**Authors:** Xia Meng, Dongsheng Wang, Guohui Liu, Mei Ding

**Affiliations:** Department of Cardiology, China-Japan Union Hospital of Jilin University, Changchun, Jilin, China

**Keywords:** case report, extracorporeal membrane oxygenation, hemodynamic instability, high-risk pulmonary embolism, percutaneous pulmonary thrombectomy, pulmonary embolism, thrombectomy, thrombolytic therapy

## Abstract

Pulmonary embolism (PE) presenting with syncope as an initial symptom is a relatively uncommon condition with a comparatively high rate of mortality (47.5%) that is prone to misdiagnosis as other cardiac and pulmonary diseases. The European Society of Cardiology classifies acute PE with persistent hypotension as high-risk PE. Patients with syncope as the initial symptom often have persistent hypotension and hypoxemia. Although traditional therapeutic approaches, such as thrombolysis and anticoagulation, have limited efficacy in treating this type of acute PE, timely use of extracorporeal membrane oxygenation (ECMO) to stabilize hemodynamics and bridging with percutaneous pulmonary thrombectomy (PPT), is an effective method in cases of high-risk PE presenting with syncope as the initial symptom. In this report, we describe the case of a 48-year-old man with no history of previous cardiac or pulmonary diseases, who had experienced sudden syncope 3 days prior to admission. Following admission, he developed cardiogenic shock [heart rate 125 beats per min, systolic blood pressure (SBP) 87 mmHg, lactate 5.2 mmol/L]. Computed tomography pulmonary angiography revealed massive bilateral PE. Although thrombolytic therapy was promptly administered, the patient continued to experience persistent hypotension (SBP 67 mmHg) and refractory hypoxemia (SaO_2_ 73%). ECMO was initiated, achieving hemodynamic stabilization within 30 min (SBP 95 mmHg, SaO_2_ 99%). Echocardiography revealed right ventricular dysfunction. To reduce pulmonary artery pressure and prevent further right heart failure, we performed emergency PPT under ECMO support. The patient was discharged on day 14 with no neurological deficits. This case highlights the efficacy of ECMO as a bridge to definitive therapy, combined with prompt PPT, in the management of high-risk PE with hemodynamic collapse. The successful outcome in this case emphasizes the importance of structured, protocol-driven hemodynamic support using ECMO and timely surgical intervention in preventing right ventricular failure.

## 1 Introduction

Following myocardial infarction and stroke, pulmonary embolism (PE) is the third most prevalent cardiovascular disease worldwide (1). According to the European Society of Cardiology, acute PE with persistent hypotension is classified as high-risk PE, with a mortality rate of approximately 25%–60%. Although PE presenting with syncope is relatively uncommon, occurring in approximately 2.2% of cases, the associated risk of mortality risk is notably high, reaching 45.7% (2, 3).

When more than 50% of the pulmonary arterial system is affected by thrombosis, there is a marked reduction in cardiac output, a reduction in arterial blood pressure, and diminished cerebral perfusion. These factors are the primary causes of syncope in PE and explain why syncope is often considered indicative of extensive PE. In addition, arrhythmias and conduction disorders resulting from excessive right ventricular load are considered major contributors to syncope in PE (4).

Whereas anticoagulation and supportive treatment are considered the main approaches for patients with PE, in cases of high-risk PE, thrombolysis and percutaneous pulmonary thrombectomy (PPT) are typically the main therapeutic options (5). In addition, for critically ill patients with unstable hemodynamics, extracorporeal membrane oxygenation (ECMO) circulatory support may sometimes be required, although given its invasiveness, the application and timing of ECMO remain controversial, particularly considering the risk of massive hemorrhage following thrombolysis (6, 7).

In this report, we describe the use of ECMO combined with PPT to treat a patient with high-risk PE presenting with syncope and hemodynamic instability.

## 2 Case presentation

**Figure 1:**
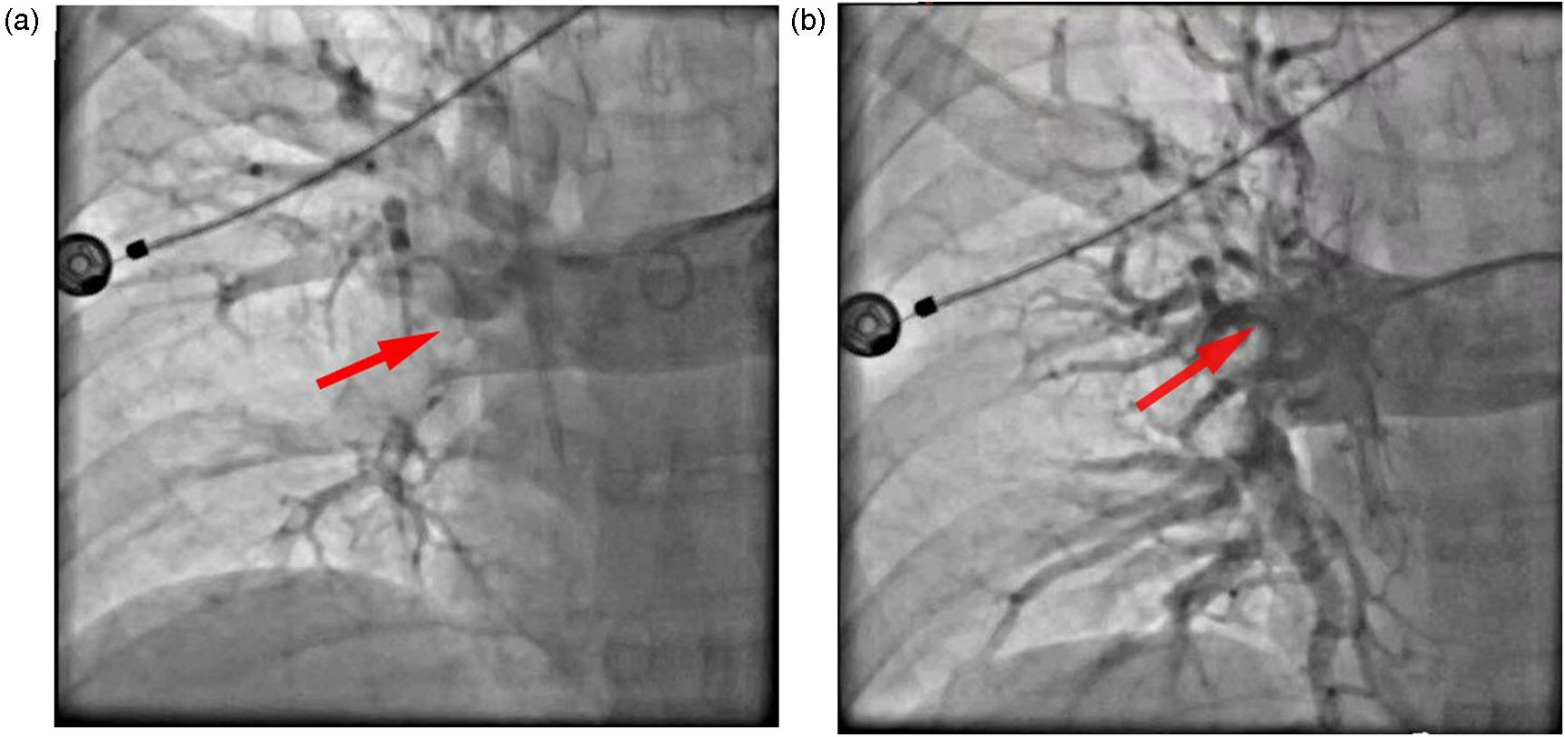
**(a)** Preoperative imaging examinations confirmed embolism of the main right pulmonary artery and its branches (arrows). **(b)** Postoperative imaging examinations confirmed that the main trunk and branches of the right pulmonary artery had all successfully re-opened (arrows).

A 48-year-old man was admitted to our hospital with persistent chest pain and shortness of breath. Three days previously, he had fainted whilst out walking. Upon admission, he lost consciousness and experienced cardiac arrest, although subsequently regained consciousness following 2 min of cardiopulmonary resuscitation. Under nasal cannula oxygen supply, his blood oxygen saturation was 82%, although he was observed to have extensive cyanosis, including the lips, accompanied by cold sweats. However, he maintained a clear consciousness and lucid speech, and there was no jugular venous distension. Although weak breath sounds were noted in both lungs, no dry or wet rales were heard on auscultation. Electrocardiography revealed sinus tachycardia (125 beats per min), inverted T waves in leads V1–V5, inverted T waves in lead III, and an incomplete right bundle branch block. Transthoracic echocardiography (TTE) revealed indirect signs suggestive of possible pulmonary embolism, including right heart enlargement, mild tricuspid regurgitation, and a widening of the pulmonary artery, with normal left ventricular systolic function. There was a reduction in right ventricular systolic function [the right ventricular/left ventricular diameter ratio (RV/LV ratio) was 1.6, the systolic displacement of the mitral valve annulus was 12 mm, and the early diastolic velocity (e') of the tricuspid valve annulus was 6 cm/s], whereas computed tomography pulmonary angiography (CTPA) revealed occlusion of the main pulmonary arteries in both lungs and several branch arteries (Figure 1a).

Despite the initiation of anticoagulatory therapy with 2 mL of heparin (12,500 IU), thrombolysis with 1.5 million IU of urokinase (UK), and ventilator-assisted ventilation, the patient's hemodynamics remained unstable. His blood pressure was 67/46 mmHg, heart rate was 110 beats per min with a regular rhythm, and blood oxygen saturation was 73%. Vasopressors were administered immediately, and having obtained consent from his family, ECMO (VA-ECMO, 3.0 L/min) was performed, and anticoagulatory treatment continued. Blood pressure was 95/70 mmHg, heart rate was 92 beats per min with a regular rhythm, and blood oxygen saturation was 99%. To prevent a further deterioration of right heart function, and the onset of cardiogenic shock (right pulmonary artery systolic pressure: 60 mmHg), PPT was performed 2 days after admission, under ECMO support. During the two days on ECMO support prior to PPT, anticoagulation was maintained with a continuous infusion of unfractionated heparin, carefully titrated to achieve a therapeutic activated partial thromboplastin time (APTT) of 1.5–2.0 times the baseline value. Postoperative imaging confirmed successful re-canalization of the right pulmonary artery trunk and branches (Figure 1b), revealing a restoration of flow compared with pre-intervention occlusion (right pulmonary artery systolic pressure: 45 mmHg; RV/LV 0.8). Throughout the postoperative period, the patient reported a dramatic improvement in his symptoms. By postoperative day 2, he stated that his breathing felt “completely normal” and he was able to hold a full conversation without shortness of breath.

**Figure 2:**
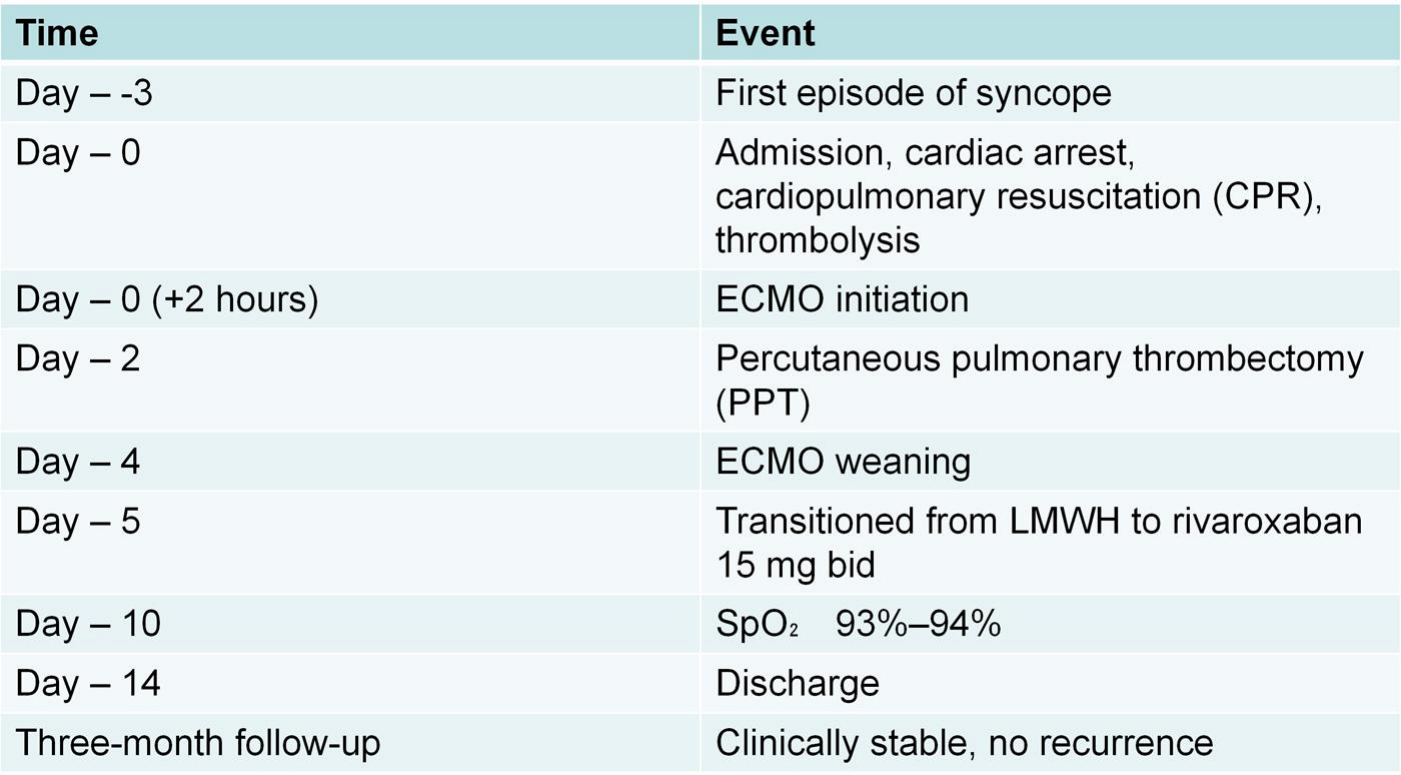
Timeline of key clinical events and interventions.

ECMO was administered before and after PPT. Following the procedure, there was a considerable improvement in the patient's hemodynamic status, with no attendant complications, such as perforation of the pulmonary artery or ventricular, being noted. Having ensured that the patient's condition had sufficiently stabilized and that blood oxygen levels had returned to normal (BP: 100/76 mmHg, SaO_2_ 94%), ECMO was withdrawn. In the immediate postoperative period, anticoagulation was initiated with subcutaneous low molecular weight heparin (1 mg/kg twice daily) to allow for careful assessment of surgical hemostasis. The patient was transitioned to oral rivaroxaban 15 mg twice daily on day 5. Ten days after admission, the patient's blood oxygen saturation had reached 93%–94% without oxygen supplementation, and following stabilization, the patient was discharged from hospital. At the three-month follow-up visit after discharge, the patient remained asymptomatic with no complaints of dyspnea, chest pain, or bleeding events. Laboratory tests revealed no evidence of coagulopathy or bleeding risk. Follow-up CTPA showed complete resolution of the previous pulmonary artery thrombus with no evidence of recurrence. We prepared a timeline figure (Figure 2) that illustrates the key clinical events and interventions.

## 3 Discussion

Although comparatively rare (∼2.2% of cases) (2), PE presenting with syncope is associated with a relatively high risk of mortality (45.7%) (3), primarily attributable to hemodynamic collapse from right ventricular failure (4). The case described herein exemplifies a high-risk PE subset, in which conventional thrombolysis failed to resolve hypoxia and hypotension, thus necessitating an escalated therapeutic approach, a scenario that is poorly addressed in the current guidelines.

Although there is currently a lack of consensus regarding the suitability of ECMO for the treatment of PE, given the risk of bleeding following thrombolysis, our findings in this case highlight the efficacy of this approach as a hemodynamic and respiratory bridge to definite therapy (6). Of note, the management of this complex, high-risk PE patient was not decided by a single physician but was the result of a formal multidisciplinary discussion involving interventional cardiology, cardiac surgery, and intensive care, a process consistent with the Pulmonary Embolism Response Team (PERT) model (8). This multidisciplinary approach is strongly supported by the recently published 2026 AHA/ACC/ACCP/ACEP/CHEST/SCAI/SHM/SIR/SVM/SVN Guideline for the Evaluation and Management of Acute Pulmonary Embolism in Adults (9). ECMO rapidly corrected the refractory hypoxia and maintained perfusion, thereby enabling safe progression to PPT, a definitive, albeit high-risk, intervention. Recent data from a large cohort study by Farmakis et al. support the use of ECMO as a bridge to reperfusion therapy in patients with high-risk PE, demonstrating improved outcomes when combined with definitive reperfusion strategies (10).

Contrary to concerns and despite concurrent thrombolysis, ECMO cannulation, and heparinization, there was no major haemorrhage, which is consistent with emerging evidence that ECMO does not inherently heighten the risk of thrombolysis-related bleeding (5). PPT performed in conjunction with ECMO support achieved immediate right ventricular afterload reduction (echo-confirmed RV/LV ratio improvement), contrasting with the delayed effects of pharmacologic thrombolysis (11). Despite syncope and hemodynamic instability, the patient's survival highlights the potential utility of early ECMO-PPT synergy with respect to improving outcomes in a cohort with a historically more than 50% likelihood of mortality. Accordingly, ECMO should be considered a first-line rescue for thrombolysis-resistant PE with RV failure.

The findings in the case challenge the prevailing dogma, which holds that ECMO should be avoided when treating thrombolized patients. We believe that PPT performed in conjunction with ECMO is a feasible option for circumventing the “thrombolysis-hemorrhage” paradox (12, 13). As a single case report, this study is inherently limited in its generalizability, and the findings should be interpreted within the context of this specific patient.

## 4 Conclusion

Currently, there remains a lack of consensus regarding the application of EMCO support treatment following thrombolysis and before PPT. However, our findings in this case highlight the potential efficacy of the early use of hemodynamic-stabilizing ECMO combined with PPT in the treatment of patients with high-risk PE, who typically have a relatively high rate of mortality associated with syncope and cardiac arrest, following ineffective thrombolytic and anticoagulant treatments. Nevertheless, there remains a need for further verification based on multi-center studies.

## Data Availability

The original contributions presented in the study are included in the article/Supplementary Material, further inquiries can be directed to the corresponding author.
